# Burnout syndrome in healthcare professionals who care for patients with prolonged disorders of consciousness: a cross-sectional survey

**DOI:** 10.1186/s12913-020-05694-5

**Published:** 2020-09-07

**Authors:** Jing Wang, Wenting Wang, Steven Laureys, Haibo Di

**Affiliations:** 1 International Unresponsive Wakefulness Syndrome and Consciousness Science Institute, Hangzhou Normal University, Hangzhou, 310036 China; 2 Coma Science Group, GIGA Consciousness, University and University Hospital of Liège, Liège, Belgium

**Keywords:** Burnout syndrome, Prolonged disorders of consciousness, Healthcare professionals, Medical area, Personality factors, Risk factors

## Abstract

**Background:**

Burnout is more common among healthcare professionals, that is an important problem of professional distress that can seriously affect healthcare professionals’ emotional state, health, medical quality and doctor-patient relationship. However, only few studies researched the burnout status of healthcare professionals who care for patients with prolonged disorders of consciousness. The aim of this study was to evaluate the level of burnout and related contributing personal and environment factors in healthcare professionals managing these patients.

**Methods:**

Institution-based cross-sectional study. Maslach Burnout Inventory-Human Services Survey was used to evaluate burnout in professionals who specially care for patients with prolonged disorders of consciousness in the neurorehabilitation department.

**Results:**

A total of 200 questionnaires were distributed, 121 were collected, among them 93 questionnaires could be used for further analysis. In this study, 61 participants (65.6%) showed burnout (55.2% physicians and 82.9% nurses). For the risks and Maslach Burnout Inventory scores, emotional exhaustion and depersonalization were correlated with age, gender, occupation, marital status, years of practice, and education level. Reduced personal accomplishment was correlated with marital status. The variables of age (< 29 years old), occupation (nurses), marital status (unmarried), years of practice (< 5 years), and educational level (≤ Undergraduate) were associated with high levels of burnout.

**Conclusions:**

Healthcare professionals who care for patients with disorders of consciousness experienced high levels of burnout. Especially those who were younger, nurse, unmarried, less practice experience or lower educational levels were more likely to experience high burnout.

## Background

Burnout syndrome is characterized by mental and emotional exhaustion, depersonalization, and a low sense of personal accomplishment [1]. Since the 1980s, scholars have carried out some studies in the field of job burnout of nurses in many medical specialties [1, 2]. This syndrome is prevalent and recognized as a major problem among healthcare professionals caring for critically ill patients, especially in the fields of psychiatry, geriatric care, emergency care, and surgical and intensive care [3]. It primarily reflects psychological pressure encountered in the work environment, which reduces the quality of life for healthcare staff and may even result in drug abuse, physical illness, depression, or death [4]. It was estimated that more than a third of healthcare professionals suffer from psychological morbidity according to specific risk factors, such as gender, marital status, age, job demands, medical specialty, and so on [5–7]. However, normally the process of burnout is slow and difficult to detect in its early stage, but it should be treated as a public health issue. Therefore, it is very important to focus on the mental health problems of healthcare professionals and evaluate risk factors for burnout in order to take preventive measures as early as possible. The Maslach Burnout Inventory-Human Services Survey (MBI-HSS) was designed to assess the frequency and intensity of perceived burnout among persons in the helping professionals in general [1, 8, 9]. It is also widely used in the study of physiological and psychological burnout of healthcare professionals [10–13].

Patients with prolonged disorders of consciousness (DOC) are those who are under the state of unconscious (coma, vegetative state/unresponsiveness wakefulness syndrome) and/or minimal conscious (minimally consciousness state). They may die or regain consciousness, or they may remain the state of unconscious or minimally conscious for longer [14]. Such patients also have the characteristics of more complications, especially infection. However, advances in medical care have led to higher survival rates and increased significantly in the number of patients with prolonged DOC. At the same time, it also brings great challenges to the healthcare staff engaged in this field. Previous studies found that family members or caregivers of patients with prolonged DOC experience significant pressure [15–17]. They are prone to job burnout, both physically and psychologically, and are subjected to stress loads that are more difficult to bear than those of the general caregivers in hospital setting. Furthermore, Gosseries et.al., [18] and Leonardi et.al., [19] found that a large number of professional caregivers suffer from moderate to low levels of burnout, especially nurses. However, there have been no other studies on burnout in healthcare professionals specifically working with prolonged DOC patients. Therefore, the main purpose of this study was to investigate the burnout levels of healthcare professionals (physicians and nurses) and to analyze the relationship between demographics and the occurrence of burnout for these professionals.

## Methods

Using a quantitative and observational study design, the data were collected by MBI-HSS scale. This cross-sectional survey was carried out using convenience sampling at neuro-rehabilitation departments for patients with prolonged DOC in 4 provinces (Shanghai, Jiangsu, Zhejiang and Henan province, China).

### Participants

The following inclusion criteria were applied: 1) the neuro-rehabilitation departments are mainly for patients with prolonged DOC after severe brain injury; 2) the healthcare professionals (physicians and nurses) working in these department, not as medical students and incoming intern; 3) the age of healthcare professionals > 18 years old; 4) the years of practice with patients with prolonged DOC ≥ one year. The healthcare professionals were excluded from participation if they felt they had too little experience caring for prolonged DOC patients. The questionnaires were given to all healthcare professionals in the seven hospitals selected. All healthcare professionals working with these patients were recruited voluntarily and anonymously in this study.

### Data collection

Data collection was carried out using the MBI-HSS scale (emotional exhaustion, depersonalization, and personal accomplishment) [1, 8], which is the most widely used measurement for evaluating burnout syndrome. The demographic information (age, gender, occupation, marital status, years of practice, working hours per day, and education level) also be collected. The MBI-HSS scale has a high reliability and validity [1, 20, 21], and the Cronbach coefficient (α) of this study is 0.86. The MBI-HSS explores emotional exhaustion, depersonalization, and the sensation of reduced personal accomplishment. The emotional exhaustion section mainly evaluates the emotional response caused by excessive work pressure, a feeling of being emotionally and physically overextended, and a loss of enthusiasm for work. The depersonalization element mainly evaluates the pressure caused by one’s attitude and feelings toward work, lack of feeling, cynical, callous, and impersonal responses toward patient care, reduced empathy, and increased cynicism. The personal accomplishment section mainly evaluates the pressure caused by the person’s view of his/her own work and feelings of competence and successful achievement; it also reflects how the person feels about the meaningfulness of his/her work. A total of 22 items from the MBI-HSS scale were used: the emotional exhaustion score included nine items with a score range of 0–54 (a score of < 19 was considered low burnout, 19–26 reflected moderate burnout, and > 26 reflected high burnout). Five items measured depersonalization with a score range of 0–30 points (< 6 reflected low burnout, 6–9 reflected moderate burnout, and > 9 indicated high burnout). The personal accomplishment evaluation included eight items with a score range of 0–48 points (> 39 reflected low burnout, 34–39 indicated moderate burnout, and < 34 reflected high burnout). All items were scored on a seven-point scale, ranging from 0 (never) to 6 (every day). The total score of each dimension was classified as low, moderate, or high. In terms of emotional exhaustion and depersonalization, persons with high scores were defined as having burnout; the higher the score, the stronger degree of burnout [1, 22–24].

### Ethics statement

The study protocols were approved by the Ethics Committee of Hangzhou Normal University. The study was conducted according to the World Medical Association’s Declaration of Helsinki. Written informed consent was obtained from the participants.

### Statistical analysis

An evaluation of descriptive statistics was performed for all demographic information. Means and standard deviations (SD) were calculated for continuous variables, while numbers and percentages were produced for categorical variables.

Analysis of variance (ANOVA) F-test comparison of score means and the least-significant difference (LSD) post-hoc analysis were used to compare the MBI-HSS scores using different variables (age, gender, occupation, marital status, years of practice, working hours per day, and education level) to determine whether there were any significant differences. About the linear-by-linear association of variables and burnout, Chi-square test and Fisher’s exact test were applied to investigate associations between variables and the presence of burnout in all participants and investigate associations between variables and the level of burnout within the MBI-HSS dimensions. Statistical significance was considered, and all statistical tests were two-sided (*p* < 0.05). All operations were done using the Statistical Package for Social Sciences (SPSS) version 20.0.

## Results

### Study and participant characteristics

A total of 200 questionnaires were distributed, and 121 were returned (60% response rate); blank and/or incomplete questionnaires (*n* = 28) were excluded from the further data analysis; and 93 valid questionnaires (46.5% effective response rate) were analyzed finally. The participants were between the ages of 20 and 62 years (34.58 ± 10.69), and the majority of them were female (60.2%), married (62.4%), physicians (62.4%). Most participants worked less than 10 h per day (72.0%). Nearly half participants had undergraduate degrees (50.5%) and one third participants had been working for less than five years (30.1%). The composition ratio of the participants’ demographic information is shown in Table 1.

**Table 1:** Composition ratio of participants’ demographic information

Characteristics	Variables	Number	Percent
Age (y)	20–29	36	38.7
	30–39	32	34.3
	40–49	14	15.1
	≥ 50	11	11.8
Occupation	Physicians	58	62.4
	Nurse	35	37.6
Gender	Male	37	39.8
	Female	56	60.2
Marital status	Married	58	62.4
	Unmarried	35	37.6
Years of practice (y)	< 5	28	30.1
	5–10	25	26.9
	10–20	15	16.1
	> 20	25	26.9
Working hours per day (h)	≤ 10	67	72.0
	> 10	26	28.0
Education level	< Undergraduate degree	27	29.1
	Undergraduate degree	47	50.5
	≥ Postgraduate degree	19	20.4

*n* number, *y* year, hour

### Burnout levels and estimated prevalence

61 participants (65.6%) showed burnout based on the MBI-HSS (i.e., the burnout was defined by high score on emotional exhaustion and/or depersonalization subscale of the MBI-HSS) (see Table 2). Among them, the prevalence of burnout among the physicians was 55.2%; the prevalence of burnout among the nurses was 82.9%. The results of statistical analysis showed that the burnout of healthcare professionals was associated with age, occupation, marital status, and years of practice. In addition, burnout was more common in those healthcare professionals with the characters of younger (< 29 years old, 80.6%), nurses (82.9%), unmarried (85.7%), or less practice working experienced (< 5 years, 82.1%).

**Table 2:** Univariate analysis of MBI-HSS scores in relation to demographics profile of the healthcare professionals

Variables	n (%)	Non-burnout, n (%)	Burnout^✝^, n (%)	χ^2^ (df)	*p* value
Total	93	32 (34.4)	61 (65.6)		
Age (y)					
20–29	36 (38.7)	7 (7.5)	29 (31.2)	13.264 (df = 3)	0.006 ** (Fisher’s exact test)
30–39	32 (34.4)	9 (9.7)	23 (24.7)		
40–49	14 (15.1)	9 (9.7)	5 (5.4)		
≥ 50	11 (11.8)	7 (7.5)	4 (4.3)		
Occupation					
Physician	58 (62.4)	26 (28)	32 (34.4)	7.413 (df = 1)	0.006 **
Nurse	35 (37.6)	6 (6.5)	29 (31.2)		
Gender					
Male	37 (39.8)	18 (19.4)	19 (20.4)	5.521 (df = 1)	0.019 *
Female	56 (60.2)	14 (15.1)	42 (45.2)		
Marital status					
Married	58 (62.4)	27 (29)	31 (33.3)	10.069 (df = 1)	0.002 **
Unmarried	35 (37.6)	5 (5.4)	30 (32.3)		
Years of practice (y)					
< 5	28 (30.1)	5 (5.4)	23 (24.7)	11.061 (df = 3)	0.011 *
5–10	25 (26.9)	7 (7.5)	18 (19.4)		
11–20	15 (16.1)	6 (6.5)	9 (9.7)		
> 20	25 (26.9)	14 (15.1)	9 (9.7)		
Working hours per day (h)					
≤ 10	67 (72.0)	22 (23.7)	45 (48.4)	0.263 (df = 1)	0.608
> 10	26 (28.0)	10 (10.8	16 (17.2)		
Education level					
< Undergraduate degree	27 (29.1)	4 (4.3)	21 (22.6)	5.839 (df = 2)	0.054
Undergraduate degree	47 (50.5)	19 (20.4)	28 (30.1)		
> Postgraduate degree	19 (20.4)	9 (9.7)	10 (10.8)		

MBI-HSS Maslach Burnout Inventory-Human Services Survey, n number, y year, h hour, χ^2^ (df) Chi-square test

^✝^High score on emotional exhaustion and/or depersonalization subscale of the MBI-HSS

*p < 0.05

**p < 0.01

Furthermore, the mean scores of each MBI-HSS subscale for all participants showed moderate emotional exhaustion (mean scores 23.22 ± 9.05), high levels of depersonalization (mean scores 11.54 ± 5.12), and highly reduced personal accomplishment (mean scores 33.46 ± 7.45). The results of ANOVA revealed that the emotional exhaustion and depersonalization of persons were significantly different in age, occupation, gender, marital status, years of practice, and education level (see Table 3). The results of ANOVA also revealed that the sensation of reduced personal accomplishment significantly differed in Marital status (F = 4.208, *p* = 0.043). For each subscale in the emotional exhaustion analysis, post-hoc testing indicated that healthcare professionals younger than 29 years had the higher score compared to those from 30 to 39 years and over 50 years old (*p* = 0.045, *p* = 0.004, respectively). Those working practice less than five years had higher scores than those working between 5 and 10 years and over 20 years (*p* = 0.036, *p* = 0.009, respectively). For those with low education level (below undergraduate degree) had higher scores than those with undergraduate degrees and postgraduate degrees (*p* = 0.002, *p* = 0.018, respectively). For the depersonalization analysis, post-hoc test algorithms indicated that healthcare professionals younger than 29 years had the higher scores compared to those aged 20–29 years, 30–39 years, and over the age of 50 (*p* = 0.014, *p* = 0.002, *p* = 0.001, respectively). Those working practice less than five years had higher scores than those working practice over 20 years (*p* < 0.001), and those working less than five years had higher scores than those working 5 to 10 years (*p* = 0.022). Those people with low education level (below undergraduate degree) had the higher score compared to those with undergraduate degrees and postgraduate degrees (*p* = 0.01, *p* = 0.001, respectively). For the personal accomplishment analysis, post-hoc testing found that healthcare professionals with low experience level (below undergraduate degree) had lower scores than those with postgraduate degrees (*p* = 0.015).

**Table 3:** Univariate analysis of MBI-HSS scores in relation to demographic profiles of the healthcare professionals

Variables	MBI-HSS subscales score								
	Emotional exhaustion			Depersonalization			Personal accomplishment		
	Mean ± SD	*p* value	Post-hoc test (*p* value)	Mean ± SD	*p* value	Post-hoc test (*p* value)	Mean ± SD	*p* value	Post-hoc test (*p* value)
Age (y)		0.018*			0.001**			0.293	
20–29	26.47 ± 9.42		20–29 vs 30–39 (0.045)	13.94 ± 5.24		20–29 vs 30–39 (0.014)	31.69 ± 6.75		
30–39	22.35 ± 7.94			11.35 ± 4.42			34.58 ± 6.38		
40–49	21.62 ± 6.36			9.15 ± 4.54		20–29 vs 40–49 (0.002)	34.31 ± 8.46		
≥ 50	17.55 ± 11.00		20–29 vs > 50 (0.004)	8.09 ± 3.21		20–29 vs > 50 (0.001)	35.36 ± 10.33		
Occupation		0.001**			< 0.001**			0.079	
Physician	20.76 ± 7.91			9.57 ± 4.37			34.52 ± 7.81		
Nurse	27.29 ± 9.44			14.8 ± 4.61			31.71 ± 6.55		
Gender		0.006**			0.012*			0.614	
Male	25.29 ± 9.04			12.61 ± 5.19			33.14 ± 6.74		
Female	20.08 ± 8.22			9.92 ± 4.62			33.95 ± 8.49		
Marital status		0.013*			< 0.001**			0.043	
Married	21.41 ± 8.66			10.1 ± 4.74			34.67 ± 8.00		
Unmarried	26.2 ± 9.00			13.86 ± 4.92			31.46 ± 6.02		
Years of practice (y)		0.037*			0.005**			0.491*	
< 5	26.61 ± 9.10			13.75 ± 5.38		< 5 vs > 20 (< 0.001)	31.68 ± 6.48		
5–10	21.48 ± 8.60		< 5 vs 5–10 (0.036)	12.08 ± 4.9		5–10 vs > 20 (0.022)	33.92 ± 6.35		
10–20	24.93 ± 7.69			10.93 ± 5.27			34 ± 8.07		
> 20	20.12 ± 9.16		< 5 vs > 20 (0.009)	8.88 ± 3.75			34.68 ± 9.03		
Working hours per day (h)		0.907			0.502			0.714	
≤ 10	23.28 ± 9.18			11.76 ± 5.19			32.79 ± 7.1		
> 10	23.04 ± 8.86			10.96 ± 4.98			35.19 ± 8.18		
Education level		0.006**			< 0.001**			0.052	
< undergraduate degree	27.81 ± 10.02			15.41 ± 4.63			31.19 ± 6.95		
undergraduate degree	21.23 ± 7.92		0.002^1^	10.38 ± 4.63		0.010^3^	33.51 ± 7.71		
≥ Postgraduate degree	21.58 ± 8.26		0.018^2^	8.89 ± 3.84		*<* 0.001^4^	36.58 ± 6.64		0.015^5^

MBI-HSS Maslach Burnout Inventory-Human Services Survey, SD standard deviation, y year, h hour

^1,2^ < undergraduate degree vs undergraduate degree: *p* = 0.002; < undergraduate degree vs ≥ postgraduate degree: *p* = 0.018

^3,4^ < undergraduate degree vs undergraduate degree: *p* = 0.010; < undergraduate degree vs ≥ postgraduate degree: *p* < 0.001

^5^ < undergraduate degree vs ≥ postgraduate degree: *p* = 0.015

* *p* < 0.05

** *p* < 0.01

**Fig. 1:**
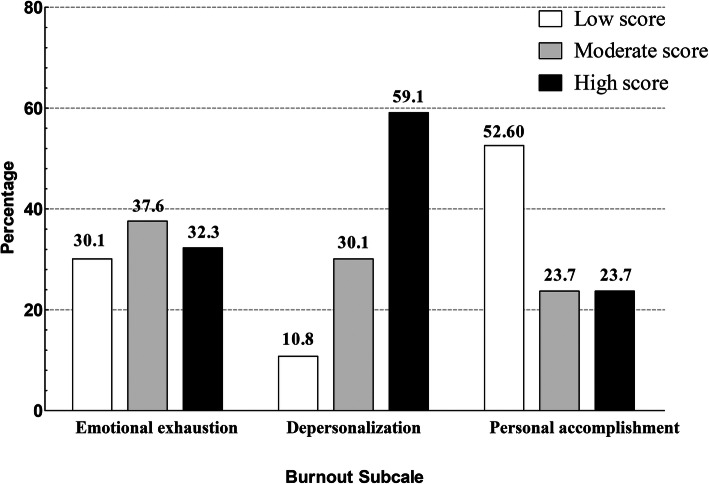
Percentage of MBI-HSS subscale score (low, moderate and high score) for healthcare professionals working with patients with prolonged disorders of consciousness

Table 4 showed the correlation between the variables and different levels of three MBI-HSS subscales (emotional exhaustion, depersonalization and personal accomplishment) among healthcare professionals. The demographic significant correlates of high emotional exhaustion were younger age (20–29 years old) (χ^2^ = 13.739, *p* = 0.027), nurses (χ^2^ = 12.799, *p* = 0.002), female (χ^2^ = 7.637, *p* = 0.022), and unmarried (χ^2^ = 9.492, *p* = 0.009). The demographic significant correlates of high depersonalization were younger age (20–29 years old) (χ^2^ = 15.231, *p* = 0.01), nurses (χ^2^ = 12.635, *p* = 0.002), unmarried (χ^2^ = 11.766, *p* = 0.003), and less working practice experience (χ^2^ = 14.851, *p* = 0.013). We did not find demographic significant correlates of lower sense of personal accomplishment in the current study. Table 4 illustrates that 28 professionals showed low score (30.1%), 35 showed moderate score (37.6%), and 30 (32.3%) showed a high score for the emotional exhaustion subscale; 10 healthcare professionals showed low score (10.8%), 28 showed moderate score 30.1%, and 55 (59.1%) showed high score for the depersonalization subscale; and 22 healthcare professionals showed high score (23.7%), 22 showed moderate score (23.7%), and 49 (52.6%) showed low score for the personal accomplishment subscale (see Fig. 1).

**Table 4:** Different levels of MBI-HSS and variables among healthcare workers

Variables	Emotional exhaustion				Depersonalization				Personal accomplishment			
	Low	Moderate	High	*p* value (χ^2^)	Low	Moderate	High	*p* value (χ^2^)	Low	Moderate	High	*p* value (χ^2^)
Age (y), n (%)				0.027* (13.739)				0.01** (15.231)				0.066 (11.34)
20–29	9 (9.7)	8 (8.6)	19 (20.4)		2 (2.2)	7 (7.5)	27 (29.0)		7 (7.5)	4 (4.3)	25 (26.9)	
30–39	11 (11.8)	14 (15.1)	7 (7.5)		3 (3.2)	7 (7.5)	21 (22.6)		8 (8.6)	11 (11.8)	13 (14.0)	
40–49	3 (3.2)	9 (9.7)	2 (2.2)		3 (3.2)	7 (7.5)	4 (4.3)		4 (4.3)	2 (2.2)	8 (8.6)	
≥ 50	5 (5.4)	4 (4.3)	2 (2.2)		2 (2.2)	6 (6.5)	3 (3.2)		3 (3.2)	5 (5.4)	3 (3.2)	
Occupation, n (%)				0.002** (12.799)								
Physician	22 (23.7)	25 (26.9)	11 (11.8)		10 (10.8)	21 (22.6)	27 (29.0)	0.002** (12.635)	16 (17.2)	16 (17.2)	26 (28.0)	0.148 (3.820)
Nurse	6 (6.5)	10 (10.8)	19 (20.4)		0 (0)	7 (7.5)	28 (30.1)		6 (6.5)	6 (6.5)	23 (24.7)	
Gender, n (%)				0.022 (7.637) *				0.195 (3.368)				0.571 (1.121)
Male	15 (16.1)	16 (17.2)	6 (6.5)		6 (6.5)	13 (14.0)	18 (19.4)		10 (10.8)	10 (10.8)	17 (18.3)	
Female	13 (14.0)	19 (20.4)	24 (25.8)		4 (4.3)	15 (16.1)	37 (39.8)		12 (12.9)	12 (12.9)	32 (34.4)	
Marital status, n (%)				0.009** (9.492)				0.003** (11.766)				0.056 (5.776)
Married	20 (21.5)	26 (28.0)	12 (12.9)		7 (7.5)	24 (3.1)	27 (29.0)		17 (18.3)	16 (17.2)	25 (26.0)	
Unmarried	8 (8.6)	9 (9.7)	18 (19.4)		3 (3.2)	4 (4.3)	28 (30.1)		5 (5.4)	6 (6.5)	24 (25.9)	
Educational level, n (%)				0.291 (2.471)				0.053 (5.281)				0.067 (5.412)
≤ Undergraduate	23 (24.7)	25 (26.9)	26 (28.0)		6 (6.5)	20 (21.5)	48 (51.6)		17 (18.3)	14 (15.1)	43 (46.2)	
≥ Postgraduate	5 (5.4)	10 (10.8)	4 (4.3)		4 (4.3)	8 (8.6)	7 (7.5)		5 (5.4)	8 (8.6)	6 (6.5)	
Years of practice (y), n (%)				0.097 (10.618)				0.013 (14.851) *				0.596 (4.672)
< 5	5 (5.4)	9 (9.7)	14 (15.1)		3 (3.2)	3 (3.2)	22 (23.7)		5 (5.4)	5 (5.4)	18 (19.4)	
5–10	12 (12.9)	7 (7.5)	6 (6.5)		2 (2.2)	6 (6.5)	17 (18.3)		4 (4.3)	8 (8.6)	13 (14.0)	
11–20	3 (3.2)	7 (7.5)	5 (5.4)		1 (1.1)	6 (6.5)	8 (8.6)		5 (5.4)	3 (3.2)	7 (7.5)	
> 20	10 (10.8)	12 (12.9)	5 (5.4)		4 (4.3)	13 (14.0)	8 (8.6)		8 (8.6)	6 (6.5)	11 (11.8)	
Educational level, n (%)				0.291 (2.471)				0.053 (5.281)				0.067 (5.412)
≤ Undergraduate	23 (24.7)	25 (26.9)	26 (28.0)		6 (6.5)	20 (21.5)	48 (51.6)		17 (18.3)	14 (15.1)	43 (46.2)	
≥ Postgraduate	5 (5.4)	10 (10.8)	4 (4.3)		4 (4.3)	8 (8.6)	7 (7.5)		5 (5.4)	8 (8.6)	6 (6.5)	
Working hours per day (h), n (%)				0.982 (0.037)				0.744 (0.583)				0.231 (2.930)
≤ 10	20 (20.5)	25 (26.9)	22 (23.7)		7 (7.5)	19 (20.4)	41 (44.1)		14 (15.1)	14 (15.1)	39 (41.9)	
> 10	8 (8.6)	10 (10.8)	8 (8.6)		3 (3.2)	9 (9.7)	14 (15.1)		8 (8.6)	8 (8.6)	10 (10.8)	

MBI-HSS Maslach Burnout Inventory-Human Services Survey, n numbers, y year, h hour

* *p* < 0.05

** *p* < 0.01

## Discussion

Burnout among healthcare professionals may affect the realization of high-quality healthcare [25]. The present study investigated the level of burnout and analyzed the risk factors leading to burnout for healthcare professionals managing patients with prolonged DOC in hospitals. Overall, the findings highlighted the high incidence of these healthcare professionals. Besides, burnout was more common in those healthcare professionals with the characters of younger, nurses, unmarried, or less practice working experienced.

Some previous studies have confirmed significantly higher risks of depression, stress, emotional exhaustion, high depersonalization, and a low sense of personal accomplishment among physicians and nurses in the general medical field [26–28]. In the present study, all healthcare professionals were responsible for the management or care of patients with prolonged DOC. The current study found that more than half of participants experienced the level of burnout, which was significantly higher than previous studies [5–7, 29]. The uniqueness of prolonged DOC patients, such as lower recovery rates, heavier physical weight, higher family pressure, higher expectations of family members [30], and some other factors, may lead to the high incidence of burnout. When analyzing the risk factors of variables, the study found that there was significant difference between different age groups, occupation groups, gender, marital status, years of practice. 82.9% nurses had the burnout, which significantly higher than physicians had. It could be attributed to the self-selection of already highly motivated physicians and resilient physicians who specialize in this challenging field of medicine; this was similar to the results of some surgeons in previous studies [31, 32]. Nurses in prolonged DOC are a special occupational group who not only need to have a wealth of professional knowledge and operational skills, but also have strong psychological qualities. Thus, the stress borne by nurses has become an occupational hazard. Extant literature showed that nurses have the greatest mental health challenges among medical professionals because they are the closest to patients and family members and are under the most pressure from patients and their families [23, 33]. The heavy workload and the lack of understanding by patients and their families could easily lead to psychological imbalances for nurses in prolonged DOC, resulting in job burnout. Moreover, the results of present study indicate that the healthcare professionals with characters of younger (< 29 years old), unmarried, and/or less practice experience are more prone to burnout syndrome in prolonged DOC field.

On the whole, healthcare professionals in prolonged DOC experienced moderate emotional exhaustion, high depersonalization, and highly reduced sense of personal accomplishment. The findings of present research were similar to the results of Hayes’s study in hemodialysis field [34], but it was significantly higher than the burnout rates of other healthcare professionals such as general practitioners and nurses [5–7]. Furthermore, this study continued to analyze the proportion of burnout level in each variable on the different dimension of MBI-HSS for healthcare professionals in prolonged DOC. Healthcare professionals with younger, female, nurses or unmarried characters were found to be more likely to experience emotional exhaustion. And high depersonalization was more likely to occur in participants with younger, female, unmarried, or less practice experience. In terms of their sense of reduced personal accomplishment, there is a clear difference between individuals with low educational levels and those with higher education levels [35]. That is, a lower level of education may be a risk factor for reduced personal accomplishment. Because, the medical healthcare professionals in medical field are generally highly educated, and the person with lower education usually cannot realize their own value. However, experienced professionals with high educational levels had less burnout, probably because they had adapted to the specialty and maintained effective coping skills [36, 37]. Therefore, professionals with low academic medical should be encouraged to further their education and improve their professional and educational level. As the result for the married status, the study found that unmarried professionals have a higher rate of burnout, so whether the support of a structure family is crucial to the psychological well-being of healthcare professionals. Preventive measures against risk factors might necessary to avoid the occurrence of burnout, such as coping skills interventions [38].

## Limitations

Due to the small number of such healthcare professionals now, this study did not meet the standard of sample size, which is one of limitations. Besides, the majority of the staff were physicians, and this could also limit the further analysis of nurses. Additionally, this study is a cross-sectional survey, and despite applying correlation statistics between variables, it does not allow to discover the causal relationship between them.

## Conclusions

Understanding job burnout and taking corresponding intervention measures are of great significance to maintain the health of healthcare professionals, reduce their turnover rate and stabilize the medical team. This study revealed that healthcare professionals who manage prolonged DOC patients experienced high levels of work-related burnout. Moreover, variables, such as age, gender, occupation, marital status, work experience, and education level, could related with the occurrence of burnout. Especially those who had these risk factors (i.e., younger, nurse, unmarried, less practice experience, or lower educational levels) were more likely to experience burnout. Hence, hospital authorities, society, and the government need to pay closer attention to these issues to improve the psychological well-being of professionals who care for prolonged DOC patients. In addition, this research provided some reference basis for the psychological adjustment and professional training of managers and medical staff [25], which is of great significance.

## Data Availability

The data sets used and /or analyzed during the current study are available from the corresponding author on reasonable request.

## Abbreviations

MBI-HSS: Maslach Burnout Inventory-Human Services Survey
DOC: Disorders of consciousness
SD: Standard deviations
LSD: Least-significant difference
ANOVA: Analysis of variance
